# Cumulative Anticholinergic Exposure and Change in Gait Speed and Grip Strength in Older Adults

**DOI:** 10.1001/jamanetworkopen.2025.19819

**Published:** 2025-07-10

**Authors:** Shelly L. Gray, Yu-Ru Su, Tesfahun C. Eshetie, Andrea Z. LaCroix, Zachary A. Marcum, Onchee Yu

**Affiliations:** 1 Department of Pharmacy, School of Pharmacy, University of Washington, Seattle; 2 Kaiser Permanente Washington Health Research Institute, Kaiser Permanente Washington, Seattle; 3 Department of Biostatistics, University of Washington, Seattle; 4 Registry of Senior Australians Research Centre, South Australian Health and Medical Research Institute, Adelaide, Australia; 5 Herbert Wertheim School of Public Health and Human Longevity Science, University of California, San Diego, La Jolla; 6 Medicus Economics, Milton, Massachusetts

## Abstract

**Importance:**

Anticholinergics have been associated with functional decline in older adults. Past studies have assumed constant effects over time and have not considered the etiologically relevant exposure window.

**Objective:**

To examine the association of anticholinergic exposure with gait speed and grip strength assuming constant and time-varying effects of daily exposure.

**Design, Setting, and Participants:**

This cohort study used data collected from February 1994 to March 2020 in the Adult Changes in Thought study at Kaiser Permanente Washington, an integrated health care delivery organization. Participants with at least 2 study visits and at least 10 years of enrollment prior to index were included. Data were analyzed from January 2023 to December 2024.

**Exposure:**

Conventional anticholinergic exposures (10-year total standardized daily dose [TSDD] and 2-year mean SDD [mSDD]) assumed constant daily exposure effects. Weighted cumulative exposures (WCE) explored different exposure windows (*T* = 2, 4, 6, 8, or 10 years) and were quantified as *T*-year weighted mSDD to allow for time-varying effects of daily exposure.

**Main Outcomes and Measures:**

Adjusted linear models with generalized estimating equations estimated mean differences (MDs) in change rates in gait speed or grip strength between anticholinergic TSDD and mSDD categories and per unit increase in weighted mSDD. Model fits were assessed by quasi-information criterion (QIC).

**Results:**

The total sample included 4283 participants, with 4210 participants (2468 women [58.6%]; mean [SD] age,74.3 [6.1] years) with 8.2 (5.4) years of follow-up in the gait speed sample, and 4200 participants (2458 [58.5%] women, mean [SD] age 74.5 [6.1]) in the grip strength sample. Compared with nonusers, a greater decline rate in gait speed was found for those with 10-year TSDD 1096 or greater (MD per year, −0.0132 [95% CI, −0.0193 to −0.0070] m/s) and for 2-year mSDD 0.5 or greater (MD per year, −0.0101 [95% CI, −0.0174 to –0.0029] m/s). The 4-year WCE model had the lowest QIC and showed a significantly greater decline rate per 1-unit increase in weighted mSDD (MD per year, −0.0034 [95% CI −0.0048 to −0.0019] m/s). There were no significant associations between conventional exposures and grip strength, but the 6-year WCE model had the lowest QIC (MD per year, −0.0329 [95% CI −0.0612 to −0.0046] kg).

**Conclusions and Relevance:**

Is this cohort study, higher anticholinergic exposure was associated with accelerated decline in physical performance, consistent with clinically meaningful decline. These findings suggest that minimizing anticholinergic medications is important for healthy aging.

## Key Points

**Question:**

Is anticholinergic exposure associated with accelerated physical performance decline in older adults?

**Findings:**

This cohort study of 4283 people found statistically significant associations for gait speed with conventional exposures (eg, use ≥1096 of total standardized daily dose in 10 years) and weighted cumulative exposures that allowed for time-varying effects, with the 4-year window fitting the data best. For grip strength, there were no significant associations with conventional exposures, but there was an association with weighted cumulative exposures, with the 6-year window fitting the data best.

**Meaning:**

This cohort study found that anticholinergics were associated with physical performance decline in older adults, supporting guidance to avoid these medications when possible.

## Teaser

This cohort study examines the association of duration and intensity of anticholinergic exposure with gait speed and grip strength in community-dwelling older adults.

## Introduction

Drugs with anticholinergic and sedative properties are frequently prescribed to older adults, with prevalence ranging from 20% to 40%.^1,2^ These medications have been associated with cognitive decline^3,4,5,6^ and falls.^7,8^ In community-dwelling older adults, higher anticholinergic and sedative burden has been associated with lower physical performance measures in cross-sectional^9,10^ and longitudinal^11,12,13,14,15,16^ studies. A meta-analysis found that anticholinergics were associated with lower gait speed compared with nonuse; however, the pooled estimated effect was small and potentially not clinically meaningful.^10^

Anticholinergics may produce acute changes in physical function through their sedative and cognitive adverse drug effects, which would likely reverse with discontinuation. However, it is not known if long-term use has sustained associations with reduced physical function.^14,15,16^ Studies have been limited by constraints of their data sources, such as defining exposure from periodic interviews, which may lead to incomplete exposure capture. Furthermore, the etiologically relevant timing of anticholinergic exposure related to physical performance decline remains unclear. Studies have assumed a constant effect of anticholinergics over time, meaning that all past anticholinergic exposures have the same effect on the current risk of physical decline. A more flexible and clinically relevant approach using the weighted cumulative exposure (WCE) method can account for varying intensity, duration, and timing of past exposures and estimates the cumulative effect size of the time-varying exposures.^17,18^ Furthermore, the WCE method can identify the etiologically relevant exposure window.^19^

Physical performance measures are associated with poor health outcomes, such as incident disability and mortality.^20,21,22^ Therefore, identifying potentially modifiable risk factors for accelerated decline in physical performance, such as medication use, is imperative for healthy aging. The objective of this study was to evaluate the association between cumulative anticholinergic exposure and change rates in gait speed and grip strength. We used WCE to allow for varying intensity, duration, and timing (hereafter, *time-varying effects*) of anticholinergic exposure, complemented with conventional exposures that assumed constant effects over time.

## Methods

This cohort study was approved by the University of Washington Human Subjects Division Institutional Review Board, and the Kaiser Permanente Interregional Institutional Review Board. All participants provided written informed consent. This study is reported following the Strengthening the Reporting of Observational Studies in Epidemiology (STROBE) reporting guideline.

### Data Source

This study used longitudinal data from the Adult Changes in Thought (ACT) study, an ongoing prospective cohort study conducted in Kaiser Permanente Washington (KPWA), an integrated health care delivery organization in Washington state.^23^ Members aged 65 years and older and without dementia at recruitment were randomly sampled and enrolled during 3 waves (ACT study cohort): 1994 to 1996, 2000 to 2003, and 2004 to 2018. Although follow-up ended March 2020, people were eligible if they had 1 follow-up visit after enrollment with a physical performance measure. Participants were assessed at baseline and biennial follow-up visits to evaluate cognitive function and physical performance and collect demographics and health information. Biennial follow-up visits ended once a participant was diagnosed with dementia. Automated data maintained at KPWA were used, including pharmacy fills, enrollment, and inpatient and outpatient diagnoses (*International Classification of Diseases, Ninth Revision *[*ICD-9*] and *International Statistical Classification of Diseases and Related Health Problems, Tenth Revision *[*ICD-10*] diagnosis codes).

### Study Design and Sample

The Figure shows the study design, outlining the timing of measurement of anticholinergics and study outcomes. We defined the index visit for a participant as their first ACT visit with an available gait speed or grip strength measure and at least 10 years of prior continuous KPWA enrollment to assess long-term anticholinergic exposure. Eligible participants had at least 1 follow-up visit with a nonmissing gait speed or grip strength value after index visit and prior to the earliest occurrence of disenrollment from KPWA or March 5, 2020. We excluded participants who had missing information on education, self-rated health, or body mass index (BMI) at index visit. eFigure 1 in Supplement 1 shows the derivation of the study samples.

**Figure.**
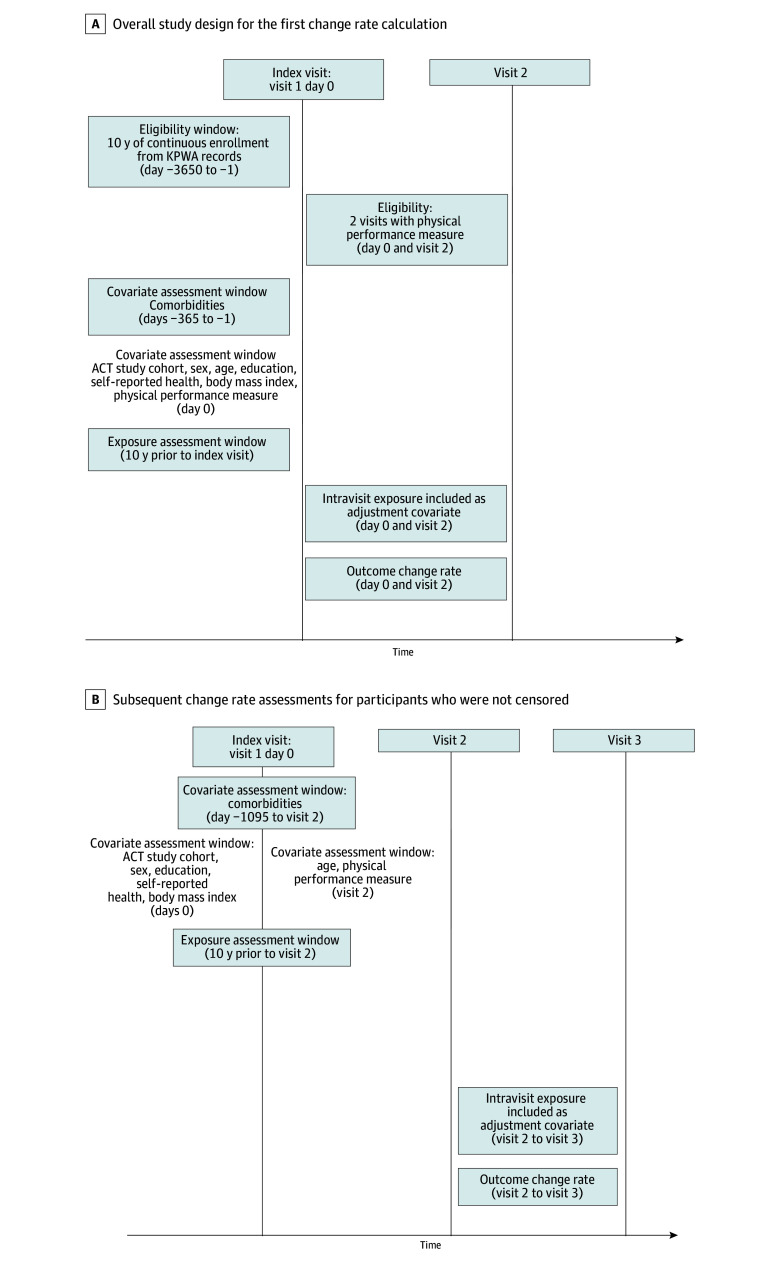
Study Design This cohort study used data from the Adult Changes in Thought (ACT) study from February 1994 to March 2020. We defined the index visit for a participant as their first visit with an available gait speed or grip strength measure and at least 10 years of prior continuous Kaiser Permanente Washington (KPWA) enrollment to assess long-term anticholinergic exposure. Eligible participants had at least 1 follow-up visit with a nonmissing gait speed or grip strength value after index visit and prior to the earliest occurrence of disenrollment from KPWA or March 5, 2020. A, Overall study design for the first change rate calculation for physical performance between the index visit (first visit, day 0) and the second visit. B, Analysis of subsequent change rate assessments for participants who were not censored. The comorbidity window extends with time in the study, where it was only available for 1 year prior to the index visit to reflect when diagnostic data became available electronically. Biennial visits occurred approximately 730 days apart but this length could vary.

### Exposures

As there is no universally accepted criterion standard measure of anticholinergics,^24^ we used strong anticholinergics from the American Geriatrics Society (AGS) Beers Criteria to focus on medications most likely associated with physical decline (eTable 1 in Supplement 1).^25,26,27^ In contrast, many tools, such as the Anticholinergic Cognitive Burden (ACB) scale, estimate overall anticholinergic burden and include medications with possible anticholinergic effects.^28^ The agents selected for this study correspond to those in the highest ACB category.

We converted prescription fills to standardized daily dose (SDD).^5,29,30^ Subsequently, we calculated the sum or mean of SDDs for all prescriptions each participant had in each exposure window of pre-specified length to obtain the total SDD (TSDD) and mean SDD (mSDD) (eMethods in Supplement 1).^5^ We evaluated a secondary exposure that combined anticholinergics and sedatives or hypnotics (eTable 1 in Supplement 1).

### Outcomes

We selected gait speed and grip strength as outcomes because they are associated with poor health outcomes in older adults.^20,21,22^ The annual change rate of each physical performance measure was defined as the difference between 2 visits, divided by the time in years between the visits.

Participants were asked to walk a 10-foot distance at their usual speed. The mean time in seconds of 2 walks was recorded. Gait speed measured in meters per second was then calculated by converting 10 feet into meters and dividing it by the mean time. For grip strength, participants were asked to use their dominant hand to grip a dynamometer (Jamar Hydraulic Hand Dynamometer) with maximal effort for 3 attempts. The mean value of the mean attempts at each visit, measured in kilograms, was used.^31^

### Covariates

Potential confounders were identified through a literature search.^5,10,14,15,32^ Covariates were ascertained from ACT study visits (age, sex, self-reported race and ethnicity, education level, BMI, self-rated health, smoking status, self-reported exercise),^33^ and KPWA electronic health records and health plan administrative data (eg, *ICD* codes, Charlson Comorbidity Index).^34^ The race categories included American Indian or Alaska Native, Asian, Black, Native Hawaiian or Pacific Islander, White, or other race (included other or multiple race). American Indian or Alaska Native and Native Hawaiian or Pacific Islander were condensed into the other race or ethnicity group to adhere to our data use agreement regarding suppression of cells with very small counts. Race and ethnicity data were collected to describe the study sample. eTable 2 in Supplement 1 illustrates how comorbidities were defined, including coronary heart disease (CHD), anxiety, Parkinson disease, insomnia, depression, and stroke.

### Statistical Analysis

Analyses were conducted at the visit level, with multiple visits from individual participants included. We considered the cumulative anticholinergic exposure that preceded each visit with an available outcome. The 2 conventional definitions of anticholinergic SDD exposure included 10-year TSDD, categorized as no use (reference group), 1-90, 91-365, 366-1095, and 1096 or greater, and 2-year mSDD, categorized as no use (reference group), less than 0.5, and 0.5 or greater. These cutpoints align with prior research.^3,5,30^ We also examined 5 WCE models that summarized the cumulative exposure in the prior 2, 4, 6, 8, and 10 years (eMethods in Supplement 1).^17^ Specifically, for a prespecified *T*-year window, we assumed a time-varying effect of anticholinergics as the multiplication of a scalar overall effect and a weight function over the *T*-year window. Under this parameterization, the time-varying effect was summarized via a weighted mSDD over the *T*-year window and its association with the outcomes (the scalar overall effect).

To assess the associations of the outcomes with each measure of anticholinergic exposure, we fit multivariable linear models at the visit level with generalized estimating equations under an independence correlation matrix to account for within-participant clustering. Robust sandwich standard error estimators were used to obtain inferences robust to the specification of correlation structure.

The adjusted models included ACT study cohort, sex, age, education, self-reported health status, and BMI at index visit. We also adjusted for physical performance measure and time-varying history of anxiety, insomnia, depression, stroke, and CHD prior to each visit. Since we were interested in the exposure that preceded the change rate, we adjusted for the mSDD of the anticholinergic exposure that occurred between the 2 visits. In the primary exposure models, we further adjusted for the TSDD of sedatives or hypnotics in this same exposure window. To determine the optimal length of the time window in the WCE analysis, we compared the quasi-information criterion (QIC) obtained from individual WCE models with 2-, 4-, 6-, 8-, and 10-year windows and selected the window with the lowest QIC value.^35^ *P* values were 2-sided, and statistical significance was set at α = .05. All analyses were performed in SAS software version 9.4 (SAS Institute). Data were analyzed from January 2023 to December 2024.

To illustrate how a weight function over the *T*-year window changes the association of exposure with the outcome, we also considered the unweighted mSDD over a 10-year period as an exposure in the model and estimated the mean difference (MD) in change rate of the outcome associated with the unweighted and weighted mSDD for 3 hypothetical anticholinergic use patterns. We provided examples of patterns of anticholinergic exposure that would result in a clinically important change for gait speed (ie, decline >0.05 m/s) under the optimal WCE models.^36,37^

## Results

The total sample included 4283 participants, with 4210 participants (2468 women [58.6%]; mean [SD] age, 74.3 [6.1] years) in the gait speed sample. The gait speed sample included 140 Asian individuals (3.3%), 142 Black individuals (3.4%), 3789 White individuals (90.0%), and 139 individuals who identified as other race (Table 1). The mean (SD) follow-up was 8.2 (5.4) years. A total of 3068 (72.9%) participants had any anticholinergic exposure. The prevalence of each medication class is provided in eTable 3 in Supplement 1.

**Table 1.** Characteristics of Participants by Anticholinergic Exposure in 10 Years Prior to Index Visit

Characteristics at index visit	Participants by anticholinergic use, No. (%)					
	Gait speed cohort			Grip strength cohort		
	All (n = 4210)	User (n = 3068)	Nonuser (n = 1142)	All (n = 4200)	User (n = 3068)	Nonuser (n = 1132)
ACT study cohort						
Enrolled in 1994-1996	2091 (49.7)	1691 (55.1)	400 (35.0)	2116 (50.4)	1714 (55.9)	402 (35.5)
Enrolled in 2000-2003	623 (14.8)	470 (15.3)	153 (13.4)	625 (14.9)	472 (15.4)	153 (13.5)
Enrolled in 2004-March 2018	1496 (35.5)	907 (29.6)	589 (51.6)	1459 (34.7)	882 (28.7)	577 (51.0)
Age, y						
Mean (SD)	74.3 (6.1)	74.7 (6.1)	73.4 (5.9)	74.5 (6.1)	74.8 (6.2)	73.5 (5.9)
65-69	1096 (26.0)	733 (23.9)	363 (31.8)	1064 (25.3)	710 (23.1)	354 (31.3)
70-74	1251 (29.7)	904 (29.5)	347 (30.4)	1247 (29.7)	898 (29.3)	349 (30.8)
75-79	984 (23.4)	746 (24.3)	238 (20.8)	981 (23.4)	751 (24.5)	230 (20.3)
80-84	591 (14.1)	458 (14.9)	133 (11.6)	611 (14.5)	474 (15.4)	137 (12.1)
≥85	288 (6.8)	227 (7.4)	61 (5.4)	297 (7.1)	235 (7.7)	62 (5.5)
Sex						
Male	1742 (41.4)	1147 (37.4)	595 (52.1)	1742 (41.5)	1154 (37.6)	1154 (37.6)
Female	2468 (58.6)	1921 (62.6)	547 (47.9)	2458 (58.5)	1914 (62.4)	544 (48.1)
Hispanic ethnicity	48 (1.1)	35 (1.1)	13 (1.1)	49 (1.2)	35 (1.1)	14 (1.2)
Race						
Asian	140 (3.3)	100 (3.3)	40 (3.5)	143 (3.4)	104 (3.4)	39 (3.4)
Black	142 (3.4)	112 (3.7)	30 (2.6)	144 (3.4)	113 (3.7)	31 (2.7)
White	3789 (90.0)	2752 (89.7)	1037 (90.8)	3774 (89.9)	2748 (89.6)	1026 (90.6)
Other, unknown, or not reported^a^	139 (3.3)	104 (3.4)	35 (3.1)	139 (3.3)	103 (3.4)	36 (3.2)
≥Some college education	2268 (53.9)	1542 (50.3)	726 (63.6)	2232 (53.1)	1517 (49.4)	715 (63.2)
BMI						
Mean (SD)	27.3 (4.9)	27.5 (5.0)	26.8 (4.6)	27.4 (4.9)	27.6 (5.0)	26.9 (4.6)
<18.5	34 (0.8)	22 (0.7)	12 (1.1)	33 (0.8)	22 (0.7)	11 (1.0)
18.5-24.9	1380 (32.8)	978 (31.9)	402 (35.2)	1367 (32.5)	969 (31.6)	398 (35.2)
25.0-29.9	1738 (41.3)	1255 (40.9)	483 (42.3)	1729 (41.2)	1255 (40.9)	474 (41.9)
≥30	1058 (25.1)	813 (26.5)	245 (21.5)	1071 (25.5)	822 (26.8)	249 (22.0)
Smoking status						
Never	2080 (49.4)	1502 (49.0)	578 (50.6)	2079 (49.5)	1503 (49.0)	576 (50.9)
Past	1927 (45.8)	1411 (46.0)	516 (45.2)	1917 (45.6)	1410 (46.0)	507 (44.8)
Current	186 (4.4)	142 (4.6)	44 (3.9)	187 (4.5)	142 (4.6)	45 (4.0)
Unknown or not reported	17 (0.4)	13 (0.4)	4 (0.4)	17 (0.4)	13 (0.4)	4 (0.4)
Fair or poor self-rated health	515 (12.2)	439 (14.3)	76 (6.7)	529 (12.6)	453 (14.8)	76 (6.7)
Regular exercise^b^	3090 (73.4)	2205 (71.9)	885 (77.5)	3061 (72.9)	2197 (71.6)	864 (76.3)
Charlson comorbidity index						
0	2786 (66.2)	1955 (63.7)	831 (72.8)	2768 (65.9)	1943 (63.3)	825 (72.9)
1	700 (16.6)	540 (17.6)	160 (14.0)	692 (16.5)	540 (17.6)	152 (13.4)
2	459 (10.9)	355 (11.6)	104 (9.1)	464 (11.0)	361 (11.8)	103 (9.1)
≥3	265 (6.3)	218 (7.1)	47 (4.1)	276 (6.6)	224 (7.3)	52 (4.6)
Comorbidities						
Depression	577 (13.7)	497 (16.2)	80 (7.0)	581 (13.8)	503 (16.4)	78 (6.9)
Anxiety	136 (3.2)	120 (3.9)	16 (1.4)	127 (3.0)	113 (3.7)	14 (1.2)
Insomnia	115 (2.7)	97 (3.2)	18 (1.6)	107 (2.5)	91 (3.0)	16 (1.4)
Parkinson disease	17 (0.4)	12 (0.4)	5 (0.4)	19 (0.5)	13 (0.4)	6 (0.5)
Coronary heart disease	696 (16.5)	571 (18.6)	125 (10.9)	712 (17.0)	585 (19.1)	127 (11.2)
Stroke	368 (8.7)	293 (9.6)	75 (6.6)	371 (8.8)	297 (9.7)	74 (6.5)
Sedative or hypnotic use in 10 y prior to index visit	1396 (33.2)	1187 (38.7)	209 (18.3)	1376 (32.8)	1171 (38.2)	205 (18.1)
Anticholinergic TSDD in 10 y prior to index visit						
Mean (SD)	NA	1281.8 (4050.8)	NA	NA	1266.3 (3937.1)	NA
1-90	NA	1376 (44.8)	NA	NA	1379 (44.9)	NA
91-365	NA	751 (24.5)	NA	NA	748 (24.4)	NA
366-1095	NA	356 (11.6)	NA	NA	359 (11.7)	NA
≥1096	NA	585 (19.1)	NA	NA	582 (19.0)	NA
Gait speed, mean (SD), meters/s	0.9 (0.2)	0.9 (0.2)	1 (0.2)	NA	NA	NA
Grip strength, mean (SD), kg	NA	NA	NA	27.4 (10.0)	26.4 (9.6)	29.9 (10.4)

Abbreviations: ACT, Adult Changes in Thought; BMI, body mass index (calculated as weight in kilograms divided by height in meters squared); NA, not applicable; TSDD, total standardized daily dose.

^a^ Other race or ethnicity included American Indian or Alaska Native, Native Hawaiian or Pacific Islander, and other races or multiple races.

^b^ Performing 1 of several listed activities for at least 15 minutes, at least 3 times per week.

### Gait Speed

The mean (SD) gait speed was 0.91 (0.24) m/s at the index visit and 0.73 (0.26) m/s at the end of follow-up. The highest 10-year TSDD category (≥1096) of anticholinergic exposure was associated with a significantly faster decline rate for gait speed compared with no use (MD per year, –0.0132 [95% CI, −0.0193 to –0.0070] m/s), while no associations were observed for the lower dose groups (Table 2). A faster decline rate in gait speed was found among participants with an mSDD of 0.5 or greater over the past 2 years (MD per year, –0.0101 [95% CI, −0.0174 to –0.0029] m/s) compared with no use, but not for those using mSDD less than 0.5. The results were similar for the secondary exposure of combined anticholinergic and sedative or hypnotic use.

**Table 2.** Associations Between Annual Change Rate in Gait Speed and Primary or Secondary Anticholinergic Exposures^a^

Exposures	Anticholinergics			Anticholinergics and sedatives or hypnotics		
	MD per year in annual change rate (95% CI), m/s	*P* value	QIC	MD per year in annual change rate (95% CI), m/s	*P* value	QIC
**Conventional exposures**						
TSDD in past 10 y						
No use	0 [Reference]	NA	16 249	0 [Reference]	NA	16 248
1-90	−0.0002 (−0.0044 to 0.0039)	.90		0.0005 (−0.0038 to 0.0047)	.83	
91-365	−0.0002 (−0.0052 to 0.0049)	.95		−0.0008 (−0.0060 to 0.0044)	.76	
366-1095	−0.0055 (−0.0119 to 0.0008)	.09		−0.0004 (−0.0068 to 0.0059)	.89	
≥1096	−0.0132 (−0.0193 to −0.0070)	<.001		−0.0105 (−0.0168 to −0.0042)	.001	
Mean SDD in past 2 y						
No use	0 [Reference]	NA	16 248	0 [Reference]	NA	16 246
<0.5	−0.0022 (−0.0062 to 0.0018)	.27		−0.0003 (−0.0042 to 0.0036)	.87	
≥0.5	−0.0101 (−0.0174 to −0.0029)	.01		−0.0079 (−0.0147 to −0.0011)	.02	
**Weighted cumulative exposures**						
Mean SDD in past 10 y, per 1-unit increase						
2 y	−0.0030 (−0.0046 to −0.0015)	<.001	16 280	−0.0036 (−0.0051 to −0.0021)	<.001	16 278
4 y	−0.0034 (−0.0048 to −0.0019)	<.001	16 075	−0.0039 (−0.0054 to −0.0025)	<.001	16 073
6 y	−0.0035 (−0.0050 to −0.0021)	<.001	16 191	−0.0042 (−0.0056 to −0.0027)	<.001	16 189
8 y	−0.0036 (−0.0051 to −0.0021)	<.001	16 505	−0.0044 (−0.0058 to −0.0029)	<.001	16 503
10 y	−0.0035 (−0.0049 to −0.0020)	<.001	16 163	−0.0042 (−0.0057 to −0.0028)	<.001	16 161

Abbreviations: MD, mean difference; NA, not applicable; QIC, quasi information criterion; SDD, standardized daily dose; TSDD, total standardized daily dose.

^a^ All models adjusted for Adult Changes in Thought study cohort, age, self-reported sex, education level (≥some college vs none), body mass index (underweight, normal, overweight, obese), self-rated health (fair or poor vs good, very good, or excellent), diagnoses of coronary heart disease, anxiety, insomnia, depression and stroke, gait speed at the first visit of change rate, and the same anticholinergic exposure between the 2 visits of change rate (mean SDD: no use, <0.5, or ≥0.5). Models with primary exposure further adjusted for TSDD of sedatives or hypnotics in the same exposure window (no use, 1-30, or ≥30).

WCEs in all 5 exposure windows were associated with a similar faster decline rate in gait speed, and the model using a 4-year window had the lowest QIC, suggesting the best fit of the data (Table 2). A 1-unit increase in the 4-year weighted mSDD was associated with a difference of −0.0034 (95% CI, −0.0048 to −0.0019) m/s per year in gait speed change rate. The estimated weight function from the 4-year WCE model demonstrated that the most proximal anticholinergic exposure had the strongest associations with the change rate (eFigure 2 in Supplement 1).

### Grip Strength

The grip strength sample included 4200 participants (2458 [58.5%] women; mean [SD] age, 74.5 [6.1] years) (Table 1) who had a mean (SD) grip strength of 27.4 (10.0) kg at the index visit and 21.8 (9.4) kg at the end of follow-up. We found no significant associations between the annual change rate in grip strength and the 2 conventional anticholinergic exposures or the combined anticholinergic and sedative or hypnotic exposure (Table 3).

**Table 3.** Associations Between Annual Change Rate in Grip Strength and Primary or Secondary Anticholinergic Exposures^a^

Exposures	Anticholinergics			Anticholinergics and sedatives or hypnotics		
	MD per year in annual change rate (95% CI), kg	*P* value	QIC	MD per year in annual change rate (95% CI), kg	*P* value	QIC
**Conventional exposures**						
TSDD in past 10 y						
No Use	0 [Reference]	NA	15 838	0 [Reference]	NA	15 837
1-90	0.0201 (−0.0540 to 0.0942)	.60		−0.0074 (−0.0874 to 0.0725)	.86	
91-365	−0.0116 (−0.1073 to 0.0841)	.81		−0.0095 (−0.1046 to 0.0856)	.85	
366-1095	0.0598 (−0.0592 to 0.1788)	.32		0.0508 (−0.0649 to 0.1664)	.39	
≥1096	−0.0414 (−0.1565 to 0.0737)	.48		−0.0313 (−0.1451 to 0.0825)	.59	
Mean SDD in past 2 y						
No Use	0 [Reference]	NA	15 838	0 [Reference]	NA	15 837
<0.5	0.0300 (−0.0473 to 0.1072)	.45		0.0630 (−0.0137 to 0.1397)	.11	
≥0.5	−0.0310 (−0.1881 to 0.1260)	.70		0.0272 (−0.1203 to 0.1746)	.72	
**Weighted cumulative exposures**						
Mean SDD in 10 y, per 1-unit increase						
2 y	−0.0334 (−0.0614 to −0.0054)	.02	15 830	−0.0324 (−0.0595 to −0.0053)	.02	15 828
4 y	−0.0298 (−0.0594 to −0.0002)	.05	15 792	−0.0322 (−0.0609 to −0.0036)	.03	15 791
6 y	−0.0329 (−0.0612 to −0.0046)	.02	15 693	−0.0360 (−0.0639 to −0.0082)	.01	15 693
8 y	−0.0384 (−0.0676 to −0.0093)	.01	16 007	−0.0406 (−0.0693 to −0.0119)	.006	16 006
10 y	−0.0367 (−0.0667 to −0.0067)	.02	15 917	−0.0393 (−0.0687 to −0.0099)	.009	15 916

Abbreviations: MD, mean difference; NA, not applicable; QIC, quasi information criterion; SDD, standardized daily dose; TSDD, total standardized daily dose.

^a^ All models adjusted for Adult Changes in Thought study cohort, age, self-reported sex, education level (≥some college vs none), body mass index (underweight, normal, overweight, obese), self-rated health (fair or poor vs good, very good, or excellent), diagnoses of coronary heart disease, anxiety, insomnia, depression and stroke, grip strength at the first visit of change rate, and the same anticholinergic exposure between the 2 visits of change rate (mean SDD: no use, <0.5, or ≥0.5). Models with primary exposure further adjusted for TSDD of sedatives or hypnotics in the same exposure window (no use, 1-30, or ≥30).

WCEs in all 5 exposure windows were significantly associated with a similar faster decline rate in grip strength, and the 6-year WCE model fit the data best (ie, lowest QIC) (Table 3). A 1-unit increase in the 6-year weighted mSDD was associated with a decline of −0.0329 (95% CI, −0.0612 to −0.0046) kg per year in grip strength. The estimated weight function under the 6-year WCE model is shown in eFigure 3 in Supplement 1.

### Time-Varying Associations With Gait Speed and Grip Strength

We examined how WCE characterized by weighted mSDD was associated with difference in change rates of gait speed between different anticholinergic patterns over 10 years (Table 4). Three use patterns were considered: no use, long-term consistent use (used 1 SDD in prior 0-4 years and 0 SDD in 4-10 years), and short-term intense use (used 4 SDD in prior 0-1 year and 0 SDD in 1-10 years).

**Table 4.** MD in Annual Change Rate in Gait Speed Estimated From Primary Anticholinergic Exposure Models

WCE model (exposure window, y)	Difference in mean SDD^a^	MD per year in annual change rate (95% CI), m/s
**Current use 1 SDD for 4 y** ^b^ ** vs no use**		
Unweighted (10)^c^	0.40	−0.0014 (−0.0020 to −0.0008)
WCE (2)	1	−0.0030 (−0.0046 to −0.0015)
WCE (4)	1	−0.0034 (−0.0048 to −0.0019)
WCE (6)	0.55	−0.0019 (−0.0044 to 0.0006)
WCE (8)	0.50	−0.0018 (−0.0042 to 0.0006)
WCE (10)	0.49	−0.0017 (−0.0042 to 0.0007)
**Current use 4 SDD for 1 y** ^**b**^ ** vs current use 1 SDD for 4 y^b^**		
Unweighted (10)^c^	0	no difference
WCE (2)	1.58	−0.0048 (−0.0138 to 0.0042)
WCE (4)	1.17	−0.0040 (−0.0130 to 0.0051)
WCE (6)	1.14	−0.0040 (−0.0131 to 0.0051)
WCE (8)	1.11	−0.0040 (−0.0129 to 0.0049)
WCE (10)	1.08	−0.0037 (−0.0121 to 0.0046)

Abbreviations: MD, mean difference; SDD, standardized daily dose; WCE, weighted cumulative exposure.

^a^ Difference in mean SDD in each comparison scenario.

^b^ TSDD for current use from unweighted estimates was 1460 (1 × 4 × 365 or 4 × 1 × 365). Mean SDD in 10 years was 1460 / (365 × 10) = 0.40 for the unweighted exposure that assumed constant effect over time. Mean SDD in 10 years for WCE was calculated by applying the estimated weight function from each WCE model, which allowed varying effects (eg, weights) over time.

^c^ The estimated association between unweighted mean SDD in 10 years per 1-unit increase and gait speed was −0.0035 (95% CI −0.0049 to −0.0021) m/s per year (*P* < .001; QIC = 16 246).

#### Long-Term Consistent Use vs No Use

The 4-year weighted mSDD was 1 for the long-term consistent use pattern, greater than 2-fold higher than the conventional 10-year unweighted mSDD (0.4). The long-term consistent use pattern was associated with an MD of −0.0034 (95% CI, −0.0048 to −0.0019) m/s of decline per year in gait speed, greater than 2-fold faster than what a model with unweighted mSDD would infer. eTable 4 in Supplement 1 illustrates the differences in 6-year weighted mSDD and the corresponding differential change rate for grip strength for the 3 use patterns.

#### Short-Term Intensive Use vs Long-Term Consistent Use

As expected, short-term intensive use and long-term consistent use corresponded to the same 10-year TSDD and conventional unweighted mSDD, and no difference in mean change rate of gait speed. However, the 4-year weighted mSDD quantified the exposure in short-term intense use greater than 2-fold higher than that in long-term consistent use (2.17 vs 1.00; difference in mean SDD, 1.17), leading to an MD of −0.0040 (95% CI, −0.0130 to −0.0051) m/s per year in gait speed decline rate for individuals with short-term intense use compared with long-term consistent use.

### Examples of Anticholinergic Use Patterns Associated With Clinically Relevant Change in Gait Speed

Using a decline of 0.05 m/sec or greater as a small but clinically important change in gait speed, we estimated effects from the 4-year WCE model for 3 high anticholinergic use patterns identified in the observed data. These results illustrate use patterns associated with this threshold of decline in 1 year (eFigure 4 in Supplement 1) and in 5 years (eFigure 5 in Supplement 1).

## Discussion

In this longitudinal cohort study of older adults, we examined the association of change rate in physical performance with anticholinergic exposures characterized in 2 conventional ways that assumed constant effects over time (categorized 10-year TSDD, and 2-year mSDD) and a set of WCEs over 5 prespecified time windows. The findings from both approaches indicate that higher anticholinergic exposure was associated with a faster decline rate in gait speed. In contrast, we found no significant associations between conventional exposures and grip strength decline rate but did find an association with WCE. Similar associations were found with the combined exposure of anticholinergics and sedatives or hypnotics. Although unweighted and weighted mSDD showed similar associations with changes in physical performance, the time-varying weights in WCE models allow differentiation between use patterns with the same total exposure—an important factor when assessing cumulative exposures over long periods.

Although the estimated annual rates of change in outcomes per 1-unit increase in weighted mSDD were small, the observed decline in gait speed associated with anticholinergic use (−0.0034 m/s per year) is comparable to the decline seen with natural aging (model estimate for 1-year increase in age was −0.0047 [95% CI, −0.0050 to −0.0045] m/s). A threshold of decline for gait speed greater than 0.05 m/s is considered clinically meaningful.^36,37^ Our findings suggest that sustained high use of anticholinergics—for example, a cumulative exposure of 1096 or greater TSDD over 5 years—could result in a gait speed decline reaching the threshold of a clinically meaningful change. Moreover, from our 4-year WCE model, we found anticholinergic use patterns that reached this threshold of decline after 1 year of intense use or after 5 years with moderate use. There is currently no consensus regarding a clinically meaningful threshold for grip strength.^38,39^ For context, each 1-year increase in age after the fifth decade of life is associated with a 0.03- to 0.4-kg decrease in grip strength.^38^ In addition, estimates for mean grip strength decline were −0.45 kg per year between ages 67 and 96 years for women and −0.95 kg per year between ages 72 and 96 years for men.^40^

To our knowledge, we provide novel findings by considering time-varying anticholinergic exposure and examining the annual change rate of physical performance (rather than score). For these reasons, our results are not directly comparable to other studies in this area that used the drug burden index (DBI) to summarize exposure to anticholinergics and sedatives.^14,15,16^ Our study results were consistent with 1 longitudinal study^14^ but not with others.^15,16^ In the Health, Aging, and Body Composition study, the area under the curve for DBI from 3 medication interviews over 5 years was associated with a significantly poorer performance in gait speed and grip strength; a 1-unit increase in the area under the curve for DBI was associated with reducing gait speed by 0.01 m/s and grip strength by 0.27 kg at year 6.^14^ In contrast, 2 large longitudinal studies that included gait speed as 1 of many functional measures found no significant association between the DBI and timed walk test.^15,16^

The comparison of results from the conventional exposures and WCE in this study highlighted some important points. We empirically selected a 10-year exposure window to capture long-term use, but the models with WCE indicated that the most etiologically relevant exposure windows were shorter, at 4 and 6 years for gait speed and grip strength, respectively. Thus, exposure that was more distal than these time windows did not contribute to the annual change rate. These results also suggested that the etiologically relevant window may vary based on the specific physical performance measures, although this requires further confirmation in other samples. Additionally, the association with daily exposure is not constant over time, and more proximal use had higher contribution to decline rate, as evidenced by the weight functions from the models with WCE.

Our study’s strengths include the use of a large, well-characterized community-based sample, a mean follow-up period of more than 8 years with a regular biennial follow-up scheme, and a computerized pharmacy database that allowed for detailed capture of long-term, daily anticholinergic exposure. We used validated, objective measures of physical performance. We evaluated exposure that occurred prior to the change rate and adjusted for exposure that occurred between visits to ensure the temporal association between exposure and outcome. The use of WCE provided a more nuanced and clinically relevant analysis of long-term exposure, which accounts for the variation of timing by incorporating differential weights for past exposures.

### Limitations

Our study has some limitations. Despite adjusting for many potential confounders, confounding by indication and unmeasured or residual confounding could bias our estimates. Anticholinergics are used for several medical conditions, and while we adjusted for some common indications (eg, depression, insomnia, anxiety), we did not adjust for all. Exposure misclassification is possible due to incomplete capture of first-generation antihistamines available over-the-counter or prescription medications filled at external pharmacies; however, this misclassification is likely to be nondifferential, and approximately 97% of KPWA enrollees obtain their medications from KPWA pharmacies.^41^ As prescribing guidance has recommended limiting anticholinergic use in older adults for several years, clinicians may have intentionally avoided these medications in older adults with multimorbidity and/or frailty, the group most likely to decline in physical performance. This would have lessened any association and may account for the small MDs in change rate. The study population was predominantly White and well educated, which may limit generalizability.

## Conclusions

In this cohort study of community-dwelling older adults, higher cumulative anticholinergic exposure was associated with greater decline rates of gait speed and grip strength beyond aging and comorbidities. The accumulation of loss over time can become clinically meaningful. Use of the WCE method demonstrated that the association between anticholinergic exposure and physical performance varied over time, with more recent exposure (within the past 4-6 years) contributing to performance decline, depending on the outcome measured. Anticholinergics are associated with numerous adverse outcomes in older adults; therefore, it is essential for clinicians to avoid their use when possible, prescribe the lowest effective dose, and periodically reevaluate patients to identify deprescribing opportunities to minimize potential harms.

## Notes

Supplement 1.
eMethods.
**eTable 1. **Medications and minimum effective dose for primary and secondary exposures **eTable 2.** Comorbidity covariate definitions **eTable 3.** Exposure by medication class during 10 years prior to index visit through the last study visit **eTable 4.** Mean difference in annual change rate in grip strength estimated from primary anticholinergic exposures models **eFigure 1.** Derivation of the study samples for gait speed (n=4210) and grip strength (n = 4200) **eFigure 2.** Estimated weight function for the model of weighted cumulative exposure and change rate in gait speed **eFigure 3.** Estimated weight function for the model of weighted cumulative exposure and change rate in grip strength **eFigure 4.** Anticholinergic use patterns associated with clinically meaningful mean change in gait speed **eFigure 5.** Long-term moderate use of anticholinergics associated with clinically meaningful mean change in gait speed

Supplement 2.
Data Sharing Statement
