# Supplementary material for: Cumulative Anticholinergic Exposure and Change in Gait Speed and Grip Strength in Older Adults
Source: JAMA Netw Open. 2025 Jul 10;8(7):e2519819. doi: 10.1001/jamanetworkopen.2025.19819 (PMC12246880; doi:10.1001/jamanetworkopen.2025.19819)
Supplement: Supplement 1. — eMethods. eTable 1. Medications and minimum effective dose for primary and secondary exposures eTable 2. Comorbidity covariate definitions eTable 3. Exposure by medication class during 10 years prior to index visit through the last study visit eTable 4. Mean difference in annual change rate in grip strength estimated from primary anticholinergic exposures models eFigure 1. Derivation of the study samples for gait speed (n=4210) and grip strength (n = 4200) eFigure 2. Estimated weight function for the model of weighted cumulative exposure and change rate in gait speed eFigure 3. Estimated weight function for the model of weighted cumulative exposure and change rate in grip strength eFigure 4. Anticholinergic use patterns associated with clinically meaningful mean change in gait speed eFigure 5. Long-term moderate use of anticholinergics associated with clinically meaningful mean change in gait speed [file jamanetwopen-e2519819-s001.pdf]

# Supplemental Online Content

Gray SL, Su Y, Eshetie TC, LaCroix AZ, Marcum ZA, Yu O. Cumulative anticholinergic exposure and change in gait speed and grip strength in older adults. *JAMA Netw Open*. 2025;8(7):e2519819. doi:10.1001/jamanetworkopen.2025.19819

**eMethods.**
**eTable 1.** Medications and minimum effective dose for primary and secondary exposures
**eTable 2.** Comorbidity covariate definitions
**eTable 3.** Exposure by medication class during 10 years prior to index visit through the last study visit
**eTable 4.** Mean difference in annual change rate in grip strength estimated from primary anticholinergic exposures models
**eFigure 1.** Derivation of the study samples for gait speed (n=4210) and grip strength (n = 4200)
**eFigure 2.** Estimated weight function for the model of weighted cumulative exposure and change rate in gait speed
**eFigure 3.** Estimated weight function for the model of weighted cumulative exposure and change rate in grip strength
**eFigure 4.** Anticholinergic use patterns associated with clinically meaningful mean change in gait speed
**eFigure 5.** Long-term moderate use of anticholinergics associated with clinically meaningful mean change in gait speed

This supplemental material has been provided by the authors to give readers additional information about their work.

**E-methods**

**Total standardized daily doses (TSDD):**

We converted prescription fills to standardized daily dose (SDD)[1,2] by multiplying the number of pills by the pill strength and then divided by the minimum effective dose per day recommended for use in older adults.[3] For each prescription fill, we created episodes of use and then calculated SDDs for each day. Subsequently, SDDs for all prescriptions each participant had in each exposure window of pre-specified length were summed or averaged to obtain the total or mean SDD (TSDD and mSDD). As example, the minimum effective dose per day for the following anticholinergic medications is oxybutynin (5 mg), tolterodine (2 mg), and paroxetine (20 mg). For each anticholinergic medication, this calculation yielded the relative unit of SDD. This exposure measure considered cumulative dose and use of multiple anticholinergic medications simultaneously. The 10-year TSDD was categorized into: non-user, TSDD of 1-90, 91-365, 366-1095 and >=1096, similar to how it was classified in our previous work.

**Weighted cumulative exposure (WCE):**

To allow heterogenous contribution of individual SDD over a given time window on the outcome of interest, we considered a weighted cumulative exposure (WCE) of anticholinergics by incorporating a time-varying weight function when summing up the SDD. The weight function, $w(s)$, at the $s^{\text{th}}$ day prior to a visit, was unknown and estimated nonparametrically as described below. It was expressed as a weighted sum $\sum_{m=1}^{M} w_m B_m(s)$ of a set of natural cubic splines $B_1(s), \dots, B_M(s)$, in which the weights $w_m$'s were unknown and estimated from the observed data.[4] We assigned 3 interior knots at the 25th, 50th, and 75th quantiles of the days over a given time window, resulting in 4 natural cubic spline functions (i.e., $M = 4$). Using the WCE over a 10-year window (or 3652 days) as an example, under this parametrization, the time-varying effect was summarized via a 10-year weighted average SDD (aSDD) and its association with the outcomes as a scalar overall effect. All parameters were estimated by fitting a multivariate linear model with Generalized Estimating Equations (GEE), assuming the following mean structure.

$$\begin{aligned}
\mu(Y_t) &= \alpha_0 + \beta_{WCE} \sum_{s=1}^{3652} w(s) SDD(t-s) + \beta_X X_t \\
&= \alpha_0 + \beta_{WCE} \sum_{s=1}^{3652} \left[ \sum_{m=1}^{M} w_m B_m(s) \right] SDD(t-s) + \beta_X X_t \\
&= \alpha_0 + \sum_{m=1}^{M} \beta_{WCE} w_m \left[ \sum_{s=1}^{3652} B_m(s) SDD(t-s) \right] + \beta_X X_t \\
&= \alpha_0 + \sum_{m=1}^{M} \gamma_m D_{m,t} + \beta_X X_t,
\end{aligned}$$

Where $D_{m,t} = \sum_{s=1}^{3652} B_m(s) SDD(t-s)$ was directly calculated from the data and $r_m = \beta_{WCE} w_m$ was estimated from GEE. With the estimations $\hat{\gamma}_m, m = 1, \dots M$, we obtained the estimated overall effect of the WCE by $\hat{\beta}_{WCE} = \sum_{m=1}^{M} \sum_{s=1}^{3652} \hat{\gamma}_m B_m(s)$ and the estimated weight function by $\widehat{w}(s) = \sum_{m=1}^{M} \widehat{w}_m B_m(s)$, where $\widehat{w}_m = \hat{\gamma}_m / \hat{\beta}_{WCE}$. We repeated the same calculations for 2-, 4-, 6- and 8-year time periods prior to each visit as well as for the secondary exposure which

included sedative/hypnotics. Under this parametrization, it is equivalent to treating the WCE as a continuous exposure and investigating its linear association with an outcome. Extending such models to incorporate categorized WCE could be challenging and non-intuitive without imposing a known weight function, which has not been established in literature regarding the impact of anticholinergics on physical function change rates.

## eTable 1. Medications and minimum effective dose for primary and secondary exposures

| | | |
|---|---|---|
| **ANTICHOLINERGICS** | | |
| Antihistamines<br>• Azatadine (2 mg)[b]<br>• Brompheniramine (12 mg)<br>• Carbinoxamine[a]<br>• Chlorpheniramine (4 mg)<br>• Clemastine (2 mg)<br>• Cyproheptadine (4 mg)<br>• Dexbrompheniramine<br>• Dexchlorpheniramine<br>• Diphenhydramine (50 mg)<br>• Doxylamine (5 mg)<br>• Hydroxyzine (75 mg)<br>• Triprolidine (10 mg)<br>Antidepressants<br>• Amitriptyline (10 mg)<br>• Amoxapine[a]<br>• Clomipramine (25 mg)<br>• Desipramine (10 mg)<br>• Doxepin (10 mg)<br>• Imipramine (10 mg)<br>• Nortriptyline (10 mg)<br>• Paroxetine (10 mg)<br>• Protriptyline (5 mg)<br>Antiarrhythmic<br>• Disopyramide (400 mg) | Antiparkinson agents<br>• Benztropine (0.5 mg)<br>• Procyclidine (7.5 mg)[b]<br>• Trihexyphenidyl (6 mg)<br>Antipsychotics<br>• Chlorpromazine (10 mg)<br>• Clozapine (20 mg)<br>• Loxapine[a]<br>• Mesoridazine (100 mg)[b]<br>• Olanzapine (2.5 mg)<br>• Pimozide (1 mg)<br>• Thioridazine (10 mg)<br>• Trifluoperazine (0.5 mg)<br>Bladder antimuscarinics<br>• Darifenacin (7.5 mg)<br>• Fesoterodine[a]<br>• Flavoxate[a]<br>• Oxybutynin<br>  o Patch (3.9 mg)<br>  o Oral (5 mg)<br>• Solifenacin (5 mg)<br>• Tolterodine (2 mg)<br>• Trospium (20 mg) | Antivertigo/antiemetic<br>• Cyclizine (50 mg)<br>• Dimenhydrinate (200 mg)<br>• Meclizine (25 mg)<br>• Prochlorperazine (15 mg)<br>• Promethazine (50 mg)<br><br>Gastrointestinal antispasmodics<br>• Atropine products (0.0582 mg)<br>• Belladonna alkaloids[c]<br>• Clidinium[b]<br>• Dicyclomine (40 mg)<br>• Glycopyrrolate (0.6 mg)[d]<br>• Homatropine (6 mg)<br>• Hyoscyamine (0.31 mg)<br>• Methscopolamine (10 mg)<br>• Propantheline (22.5 mg)<br>• Scopolamine<br>  o Patch (0.33 mg)<br>  o Oral (0.0195 mg)<br><br>Skeletal muscle relaxants<br>• Cyclobenzaprine[a]<br>• Orphenadrine[a] |
| **SEDATIVE/HYPOTICS** | | |
| Benzodiazepines<br>• Alprazolam (0.25 mg)<br>• Chlordiazepoxide (10 mg)<br>• Clonazepam (0.5 mg)<br>• Clorazepate (7.5 mg)<br>• Diazepam (1 mg)<br>• Flurazepam (15 mg)<br>• Lorazepam (0.5 mg)<br>• Oxazepam (20 mg)<br>• Temazepam (7.5 mg)<br>• Triazolam (0.125 mg) | Z-drugs<br>• Eszopiclone (1 mg)<br>• Zaleplon (5 mg)<br>• Zolpidem tartrate (5 mg)<br><br>Others<br>• Ramelteon (8 mg)<br>• Chloral hydrate (250 mg)<br>• Meprobamate (600 mg) | |

[a]Minimum effective doses are only given for medications used by participants in the cohort

[b]Medications used by participants but no longer on the market; mesoridazine and clidinium were on the Beers list for highly anticholinergic medications

[c]1 suppository=1 SDD

[d]Not on Beers List but a pharmacological effect is antagonism of muscarinic receptors.

## eTable 2. Comorbidity covariate definitions

| Comorbidity[a] | ACT interview data | ICD-9 and ICD-10 Codes |
|---|---|---|
| Coronary heart disease | self-reported physician diagnosis of coronary heart disease | |
| Depression | 10-item Center for Epidemiological Studies Depression (CESD-10) score ≥10 **OR** diagnostic codes | ICD-9: 296.2x, 296.3x, 311<br>ICD10: F32.xx, F33.xx |
| Anxiety | | ICD-9: 300.00, 300.01, 300.02, 300.09<br>ICD-10: F41.9, F41.0, F41.1, F41.8 |
| Insomnia | | ICD-9: 780.50, 780.51, 780.52, 327.00-327.09<br>ICD-10: G47.9, G47.30, G47.00, G47.01, G47.04, G47.05, G47.09 |
| Parkinson disease | | ICD-9: 332.0<br>ICD-10: G20 |
| Stroke | self-reported prior diagnosis of stroke, transient ischemic attack or carotid endarterectomy **OR** diagnostic codes | ICD-9: 431.x, 434.x, 438.x<br>ICD-10: 160.9, I63.xx, 169.3xx |

ICD = International Classification of Diseases.
[a] ICD diagnosis codes 1 year prior to index visit to end of follow-up were used to derive these covariates. Comorbidities were included in the models as time-varying and updated prior to each change score.

## eTable 3. Exposure by medication class during 10 years prior to index visit through the last study visit

| | Gait Speed Cohort (n=4210) | | | | Grip Strength Cohort (n=4200) | | | |
|---|---|---|---|---|---|---|---|---|
| Medication class | No. (%) Participants | Total TSDD filled | Anticholinergics % of total TSDDs[a] | Anticholinergics and sedatives or hypnotics % of total TSDDs[b] | No. (%) Participants | Total TSDD filled | Anticholinergics % of total TSDDs[c] | Anticholinergics and sedatives or hypnotics % of total TSDDs[d] |
| Antihistamines | 2407 (57.2) | 1019416 | 14.5 | 12.0 | 2412 (57.4) | 1045822 | 14.8 | 12.2 |
| Gastrointestinal antispasmodics | 1775 (42.2) | 346176 | 4.9 | 4.1 | 1771 (42.2) | 353619 | 5.0 | 4.1 |
| Antivertigo/antiemetic | 1607 (38.2) | 83082 | 1.2 | 1.0 | 1619 (38.5) | 93421 | 1.3 | 1.1 |
| Antidepressants | 1529 (36.3) | 4627384 | 66.0 | 54.4 | 1534 (36.5) | 4632690 | 65.3 | 53.4 |
| Bladder antimuscarinics | 731 (17.4) | 878850 | 12.5 | 10.3 | 747 (17.8) | 913278 | 12.9 | 10.6 |
| Antipsychotics | 26 (0.6) | 23768 | 0.3 | 0.3 | 26 (0.6) | 23768 | 0.3 | 0.3 |
| Antiarrhythmic | 21 (0.5) | 30019 | 0.4 | 0.4 | 20 (0.5) | 27457 | 0.4 | 0.3 |
| Antiparkinson agents | 13 (0.3) | 1585 | 0.02 | 0.02 | 14 (0.3) | 1865 | 0.03 | 0.02 |
| Benzodiazepines | 1759 (41.8) | 1397322 | NA | 16.4 | 1742 (41.5) | 1403241 | NA | 16.3 |
| Z-drugs | 247 (5.9) | 67551 | NA | 0.8 | 244 (5.8) | 65735 | NA | 0.8 |
| Other sedatives | 248 (5.9) | 36916 | NA | 0.4 | 250 (6.0) | 36844 | NA | 0.4 |

NA, not applicable; TSDD, total standardized daily dose

[a]Total of 7010280 TSDDs
[b]Total of 8512070 TSDDs
[c]Total of 7091920 TSDDs
[d]Total of 8597740 TSDDs

## eTable 4. Mean difference in annual change rate in grip strength estimated from primary anticholinergic exposures models

| WCE model (exposure window, y) | Difference in mean SDD[a] | MD per year in annual change rate (95% CI) kg |
|---|---|---|
| **Current use 1 SDD for 4 y[b] vs no use** | | |
| Unweighted (10)[c] | 0.40 | -0.0148 (-0.0273, -0.0023) |
| WCE (2) | 1 | -0.0334 (-0.0614, -0.0054) |
| WCE (4) | 1 | -0.0298 (-0.0594, -0.0002) |
| WCE (6) | 0.33 | -0.0109 (-0.0734, 0.0515) |
| WCE (8) | 0.87 | -0.0333 (-0.0873, 0.0206) |
| WCE (10) | 0.93 | -0.0343 (-0.0923, 0.0237) |
| **Current use 4 SDD for 1 year[b] vs current use 1 SDD for 4 years[b]** | | |
| Unweighted (10)[c] | 0 | no difference |
| WCE (2) | -3.75 | 0.1252 (-0.1479, 0.3982) |
| WCE (4) | -4.74 | 0.1414 (-0.0839, 0.3667) |
| WCE (6) | -4.19 | 0.1380 (-0.0913, 0.3674) |
| WCE (8) | -2.16 | 0.0830 (-0.1466, 0.3127) |
| WCE (10) | -0.16 | 0.0057 (-0.1858, 0.1972) |

Abbreviation: MD, mean difference, SDD, standardized daily dose; WCE, weighted cumulative exposure.

[a] Difference in mean SDD in each comparison scenario.

[b] TSDD for all current use was 1460 (1 × 4 × 365 or 4 × 1 × 365). Mean SDD in 10 years was 1460/(365 × 10) = 0.40 for the unweighted exposure that assumed constant effect over time. Mean SDD in 10 years for WCE was calculated by applying the estimated weight function from each WCE model, which allowed varying effects over time.

[c]The estimated association between unweighted mean SDD in 10 years per 1-unit increase and grip strength was -0.0370 (95% CI -0.0681 to -0.0059) kg per year (p <.001; QIC 15836).

**eFigure 1.** Derivation of the study samples for gait speed (n=4210) and grip strength (n=4200)

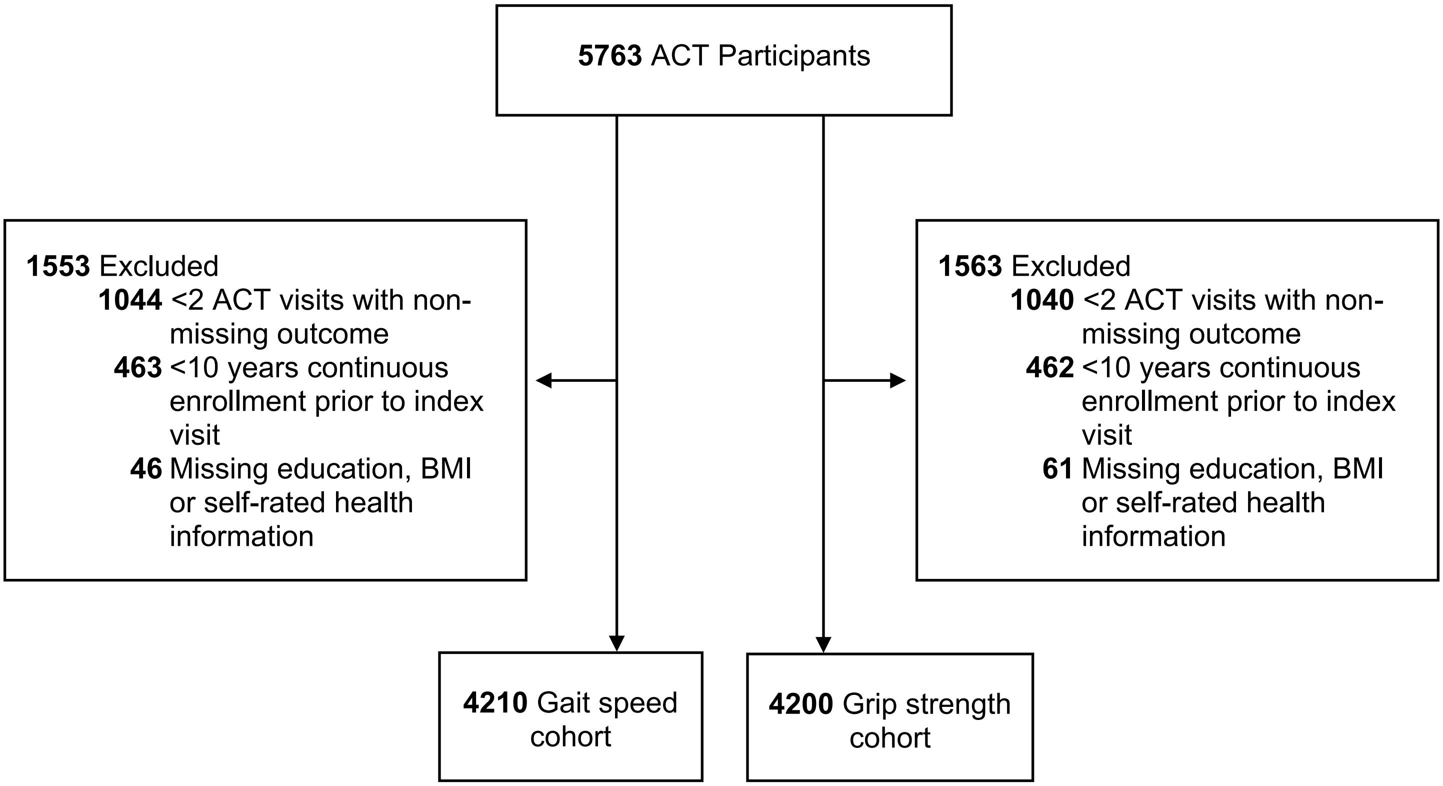

**eFigure 2. Estimated weight function for the model of weighted cumulative exposure and change rate in gait speed.** The weight function is represented by the solid curve with the 95% confidence interval in dotted curves. The model was of the weighted cumulative exposure of primary anticholinergic exposure in 4 years prior to visits and its association with change rate in gait speed.

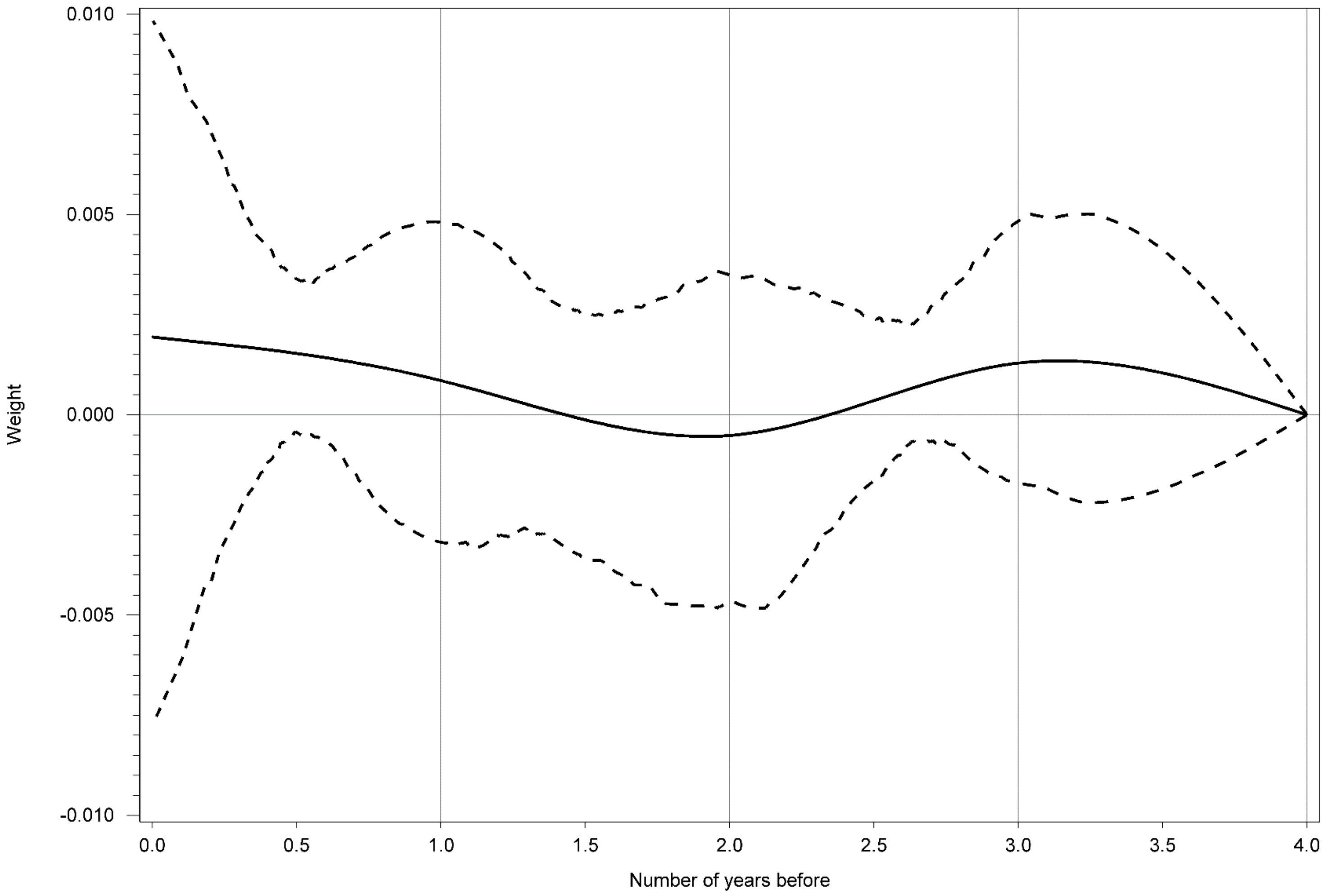

**eFigure 3. Estimated weight function for the model of weighted cumulative exposure and change rate in grip strength.** The weight function is represented by the solid curve with the 95% confidence interval in dotted curves. The model was of the weighted cumulative exposure of primary anticholinergic exposure in 6 years prior to visits and its association with change rate in grip strength.

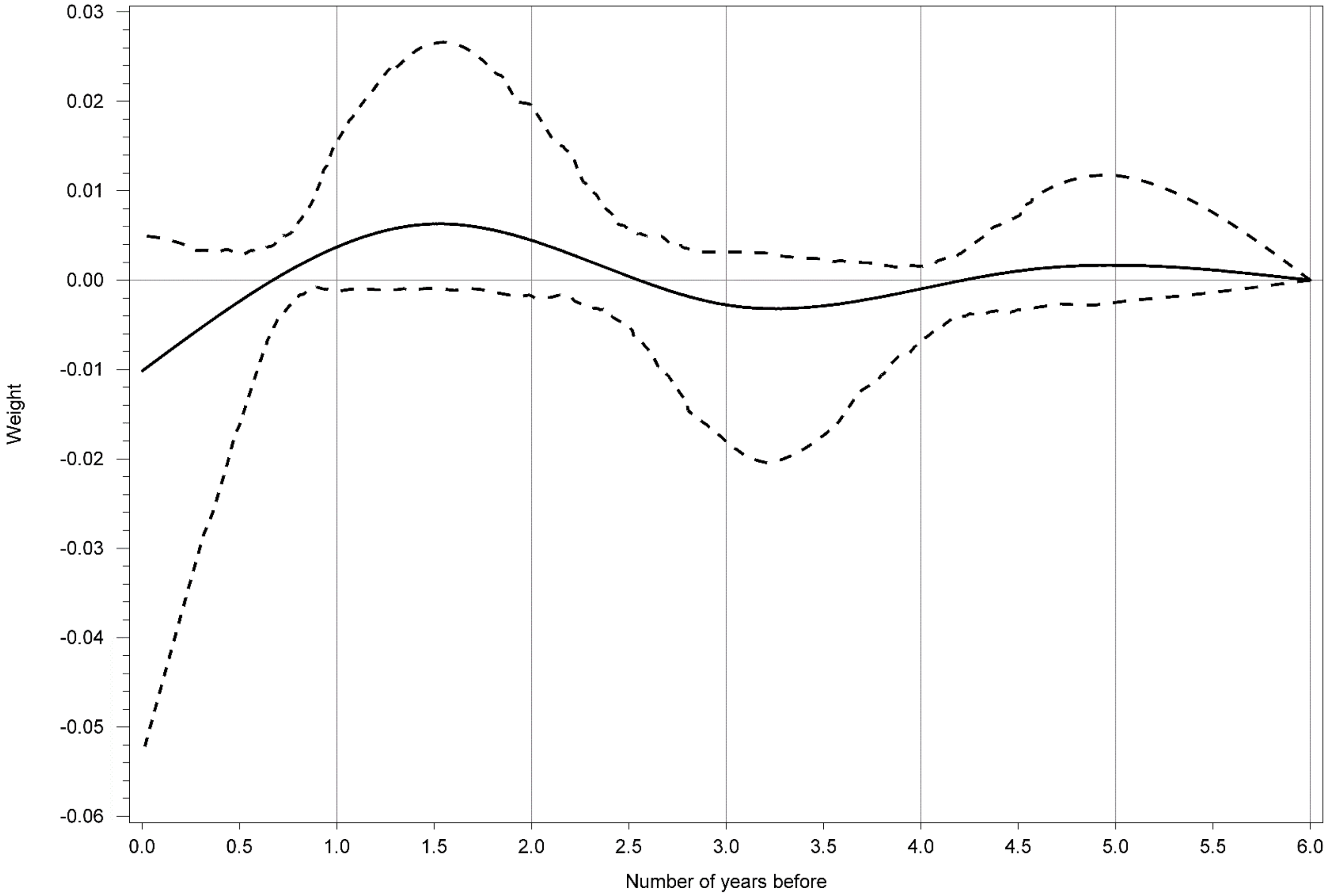

**eFigure 4. Anticholinergic use patterns associated with clinically meaningful mean change in gait speed.** Each panel illustrates a pattern of use, the weighted 4-year mean SDD and the estimated annual change in gait speed. A) periodic intense use: 2 periods with 40 SDD for 6 months (40, 0, 0, 0, 0, 0, 0, 40), weighted 4-year mean SDD = 16.97, estimated annual change = -0.058 m/sec. B) periodic intense use: 4 periods with 40 SDD for 3 months within every year, weighted 4-year mean SDD = 16.13, estimated annual change = -0.055 m/sec.

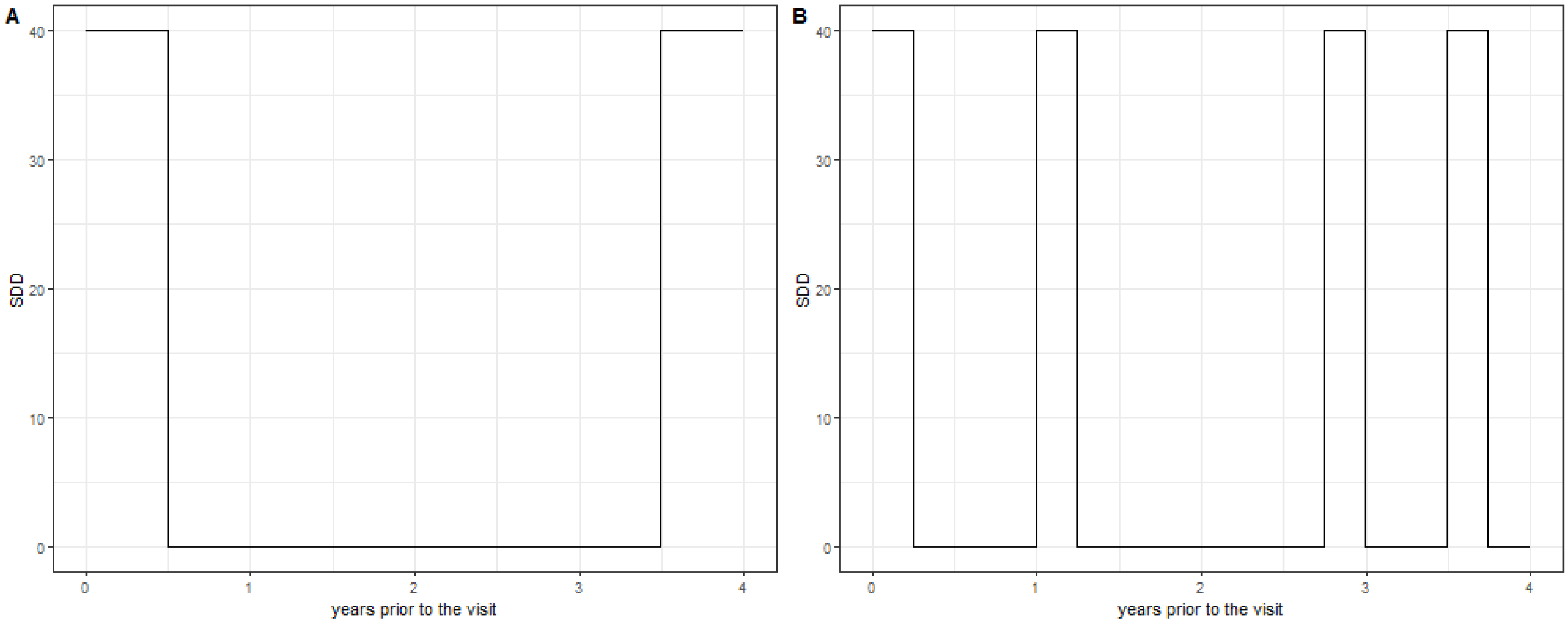

**eFigure 5. Long-term moderate use of anticholinergics associated with clinically meaningful mean change in gait speed.** This figure illustrates a pattern of long-term moderate anticholinergic use with 3 SDD for 9 years, corresponding to a weighted mean SDD of 3 for each rolling 4-year window and the estimated change over 5 years of -0.051 m/sec.

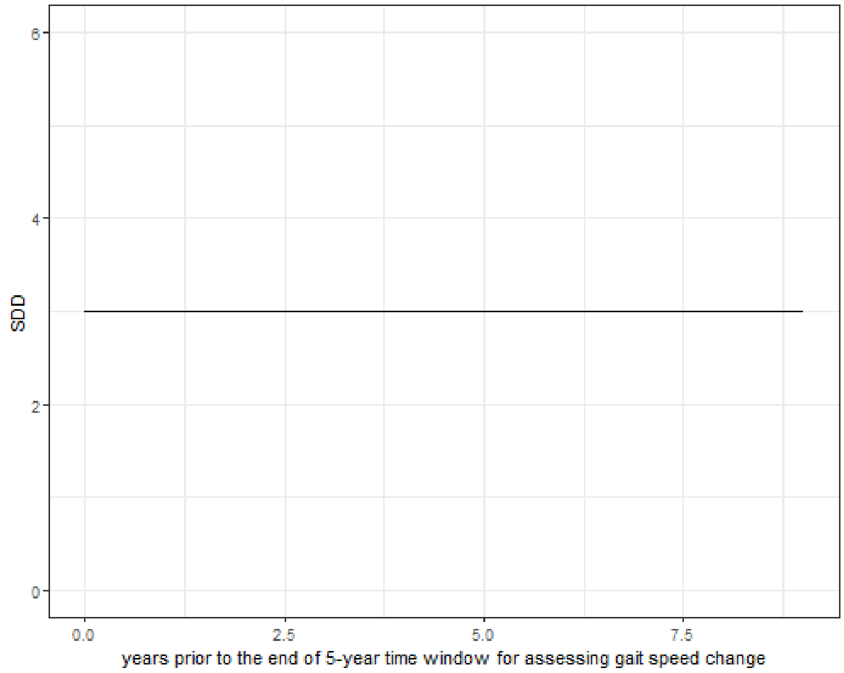


© 2025 Gray SL et al. *JAMA Network Open*.
